# Loss of Dok-3 in Non-tumor Cells Induces Malignant Transformation of Benign Epithelial Tumor Cells of the Intestine

**DOI:** 10.1158/2767-9764.CRC-22-0347

**Published:** 2022-12-08

**Authors:** Sumimasa Arimura, Akane Inoue-Yamauchi, Kotoe Katayama, Tatsuo Kanno, Hiroki Jozawa, Seiya Imoto, Yuji Yamanashi

**Affiliations:** 1 Division of Genetics, The Institute of Medical Science, The University of Tokyo, Tokyo, Japan.; 2 Laboratory of Sequence Analysis, Human Genome Center, The Institute of Medical Science, The University of Tokyo, Tokyo, Japan.; 3 Division of Health Medical Intelligence, Human Genome Center, The Institute of Medical Science, The University of Tokyo, Tokyo, Japan.

## Abstract

The fundamental difference between benign and malignant tumors lies in their invasive ability. It is believed that malignant conversion of benign tumor cells is induced by a tumor cell–intrinsic accumulation of driver gene mutations. Here, we found that disruption of the *Dok-3* tumor suppressor gene led to malignant progression in the intestinal benign tumor model ApcMin/+ mice. However, *Dok-3* gene expression was undetectable in epithelial tumor cells and the transplantation of bone marrow cells lacking the *Dok-3* gene–induced malignant conversion of epithelial tumor cells in ApcMin/+ mice, indicating a previously unrecognized tumor cell–extrinsic mechanism. Moreover, the Dok-3 loss–induced tumor invasion in ApcMin/+ mice required CD4^+^ and CD8^+^ T lymphocytes, but not B lymphocytes. Finally, whole-genome sequencing showed an indistinguishable pattern and level of somatic mutations in tumors irrespective of the *Dok-3* gene mutation in ApcMin/+ mice. Together, these data indicate that Dok-3 deficiency is a tumor-extrinsic driving force of malignant progression in ApcMin/+ mice, providing a novel insight into microenvironments in tumor invasion.

**Significance:**

This study uncovers tumor cell–extrinsic cues that can induce malignant conversion of benign tumors without intensifying mutagenesis in tumors, a novel concept potentially providing a new therapeutic target in malignancy.

## Introduction

Benign tumors become malignant when they acquire the ability to breach epithelial basement membrane and invade into neighboring tissues, which is the first essential step of the invasion-metastasis cascade followed by intravasation, extravasation, formation of micrometastases, and colonization of distant organs. Metastasis is estimated to be responsible for around 90% of cancer-associated mortality, yet the underlying mechanisms of the invasion-metastasis cascade remain poorly understood (1).

It is widely accepted that accumulation of genetic alterations in driver genes, including oncogenes and tumor suppressor genes, causes tumorigenesis and tumor malignant progression, a multistep carcinogenic process initially documented in colorectal cancer (2). Emerging evidence suggests that the tumor cell–intrinsic gene mutations promote carcinogenesis not only by affecting tumor cells but also by shaping the tumor microenvironment (TME), which comprises activated and/or recruited non-tumor stromal cells such as fibroblasts, endothelial cells, lymphocytes, macrophages, and other types of inflammatory cells (3, 4). For example, the loss of the tumor suppressor Smad4 in tumor epithelial cells mutated in *Apc* promotes the invasiveness of intestinal tumors through recruitment of matrix metalloproteinase (MMP)-expressing CCR1^+^ bone marrow–derived cells to the invasion front (5).

Recent studies have further shown that even stromal cells can induce development and malignant progression of tumors by modulating neighboring epithelial and/or tumor cells. For example, glutathione peroxidase 4 (Gpx4) deficiency in myeloid cells induces tumorigenesis in various tissues and malignant progression of colorectal tumors through reactive oxygen species–mediated epithelial and/or tumor mutagenesis, including mutations in the driver genes: *Apc*, *kras*, and *Tp53* (6), indicating stromal cell–dependent mechanisms underlying the epithelial and/or tumor cell–intrinsic mutations that cause tumor development and malignant progression. Similarly, Tgfbr2 deficiency in fibroblasts has been shown to induce prostate intraepithelial neoplasia and forestomach squamous cell carcinoma (7). Moreover, the loss of Smad4 or Lkb1 in T cells resulted in gastrointestinal carcinomas or polyps, respectively (8, 9). However, the involvement of epithelial mutagenesis in the above examples of stromal cell–driven tumor development remains unclear.

The Dok family of adaptor proteins consists of seven members that can be classified into three subgroups based on their structural similarities and expression patterns (10). Dok-1, -2, and -3 compose a subgroup, and are key negative regulators of hematopoietic growth and survival signaling. These Dok family proteins cooperatively inhibit macrophage proliferation and indeed Dok-1*/*Dok-2/Dok-3 triple knockout mice on a C57BL/6 and 129/Sv mixed background develop histiocytic sarcoma, an aggressive malignancy of macrophages (11). In addition, on a 129S1/SvImj genetic background, single, double, or triple deficiency in *Dok1/2/3* genes causes lung cancer in mice (12), together indicating tumor suppressor function. However, the role of Dok-1/-2/-3 in malignant conversion of benign tumors remains unknown. Therefore, we employed the intestinal benign tumor model ApcMin/+ mice (13, 14) in which malignant progression has been well studied by adding mutations to tumor epithelial cells mutated in *Apc* (15).

In the current study, by using ApcMin/+ mice lacking Dok-1/-2 or Dok-3, we show that Dok-1/-2 and Dok-3 play distinctive roles in growth and malignant progression of intestinal tumors. Intriguingly, the loss of Dok-3 induced malignant progression in ApcMin/+ mice without significant stimulation of gene mutations in tumors. In addition, transplantation of Dok-3–deficient bone marrow cells also induced malignant progression in ApcMin/+ mice, indicating a novel tumor cell–extrinsic mechanism underlying the malignant conversion of benign tumors.

## Materials and Methods

### Mice

All experiments involving animals were approved by the Animal Ethics Committee of The Institute of Medical Science, The University of Tokyo (A21-21; Tokyo, Japan). ApcMin/+ mice (C57BL/6J-*Apc^Min^*/J: stock no. 002020) and Csf1^op^ mice (B6;C3Fe *a/a-Csf1^op^*/J: stock no. 000231) were obtained from Jackson Laboratory. Rag1 knockout (stock no. 002216), Cd4/8 knockout (stock no. 002664), Tcrd knockout (stock no. 002120), and Igh6 knockout (stock no. 002399) mice were obtained from Dr. Hiroshi Kiyono (The University of Tokyo, Tokyo, Japan) through the Jackson Laboratory. Dok-3 knockout mice on a C57BL/6 and 129/Sv mixed background were provided by Dr. Brian Seed (16), which had been backcrossed with C57BL/6 at least 12 times. Dok-1/-2 knockout mice on a C57BL/6J background were described previously (17). Mice from 6 to 7 months old were used in this study.

### Histopathological Analysis, IHC, and Immunofluorescence

For histopathological analysis, tissues were fixed with 4% paraformaldehyde, paraffin embedded, and serially sectioned at 4 μm thickness. The paraffin sections were stained with hematoxylin and eosin (H&E). H&E-stained sections were analyzed using an optical microscope CX-33 (Olympus). Histologic assessment was reviewed in consultation with a pathologist. For IHC, paraffin sections prepared at 4 μm thickness were deparaffinized, rehydrated, and subjected to antigen retrieval by heating the sections at 90°C in HistoVT One (Nacalai Tesque). After inactivating endogenous peroxidases with 3% hydrogen peroxide and blocking in 2.5% normal goat or horse serum, the sections were incubated with a primary antibody against Ki67 (NeoMarkers), E-Cadherin, or CD3ε (Cell Signaling Technology). ImmPRESS Reagent Anti-Rabbit IgG and ImmPACT DAB (Vector Laboratories) were used for detection. After counterstaining with hematoxylin, the slides were mounted. For immunofluorescence, tissues were fixed in 4% paraformaldehyde, optical cutting temperature-embedded, snap-frozen in liquid nitrogen, and sectioned at 6 μm thickness. The frozen sections were incubated with the primary antibodies specific for CD45, CD11b, Gr1, B220 (BioLegend), Cytokeratin, or α-smooth muscle actin (SMA; Abcam), followed by Alexa Fluor 488–conjugated or Alexa Fluor 594–conjugated goat secondary antibodies (Molecular Probes/Thermo Fisher Scientific). After counterstaining with 4ʹ,6-diamidino-2-phenylindole (DAPI), the slides were mounted. All bright-field and fluorescence images were captured using a BZ-9000 microscope (Keyence). The mean Ki67-labeling indices were calculated as the number of Ki67-positive cells per total number of tumor cells by counting at ×120 in five independent microscopic fields per tumor (*n* = 3).

### Bone Marrow Transplantation

Nucleated bone marrow cells were collected from 8-weeks-old wild-type (WT) and Dok-3 knockout mice and intravenously injected into lethally irradiated ApcMin/+ mice (abbreviated as *Apc* mice). Irradiation was performed using an IBL-437C instrument (^137^Cs, CIS Bio-International) at 9.5 Gy.

### Quantitative and Semiquantitative RT-PCR

Total RNA was extracted from cells, tumors (*n* = 3 for *Apc* mice or ApcMin/+;Dok-3 knockout mice, abbreviated as *Apc/Dok3* mice), or organoids (*n* = 3; generated as described previously; ref. 18) with ISOGEN (Nippon Gene) according to the manufacturer's instruction. cDNA was synthesized with PrimeScript RT reagent kit (Takara), and qRT-PCR analysis was carried out on a CFX Connect Real-Time PCR Detection System (Bio-Rad) using TB Green Premix Ex Taq II (Tli RNaseH Plus; Takara) and the following primer sets: *Dok3* (F: TTTGGCAAGAAATGCTGGCG, R: CCCTGTGGACCTGTCGCCTG) and *Gapdh* (F: ATGGTGAAGGTCGGTGTGAACG, R: CGCTCCTGGAAGATGGTGATGG), *Il1b* (F: CTTGTGCAAGTGTCTGAAGCAG, R: AGGTCAAAGGTTTGGAAGCAGC), *Il6* (F: ACCACTTCACAAGTCGGAGGC, R: CTGCAAGTGCATCATCGTTGTT), *Il7a* (F: ACCTCAACCGTTCCACGTC, R: GCTTTCCCTCCGCATTGAC), *Cxcl1* (F: TCCAGAGCTTGAAGGTGTTGCC, R: AACCAAGGGAGCTTCAGGGTCA), *Cxcl2* (F: CATCCAGAGCTTGAGTGTGACG, R: GGCTTCAGGGTCAAGGCAAACT), *Cxcl10* (F: ATCCTGCTGGGTCTGAGTGG, R: AGGATAGGCTCGCAGGGATG), *Ccl11* (F: TCCATCCCAACTTCCTGCTGCT, R: CTCTTTGCCCAACCTGGTCTTG), *Tnfa* (F: CCACGTCGTAGCAAACCACC, R: GACAAGGTACAACCCATCGGC), *Cox-2* (F: TCCCGTAGCAGATGACTGCC, R: CCTTGGGGGTCAGGGATGAA). Semiquantitative RT-PCR analysis was performed with GoTaq (Promega) and the following primer sets: *Dok1* (F: TTGGAGATGCTGGAGAATTCGC, R: AGTCAGTTCTGAGGATATCCTG), *Dok2* (F: AGTGACTGGATACAGGCCATC, R: AGCAATGACCTTTTCTAAGGC), *Dok3* (F: TTTGGCAAGAAATGCTGGCG, R: TCCATGGGAACAAAGCCCCT), *Gapdh* (F: GCACCACCAACTGCTTAGCC, R: CCATCACGCCACAGCTTTCC).

### Flow Cytometry

Mononuclear cells from size-matched pooled intestinal tumors (containing five tumors ≥2 mm in diameter) of 6-month-old *Apc* mice or *Apc/Dok3* mice (*n* = 6 for each genotype) were stained with the following fluorochrome-conjugated antibodies (BioLegend): allophycocyanin (APC)-conjugated anti-CD3ε (145-2C11); APC/Fire 750-conjugated anti-CD11c (N418); Alexa Fluor 488–conjugated anti-Foxp3 (MF-14); Alexa Fluor 700–conjugated anti-F4/80 (BM8); Brilliant Violet (BV421)-conjugated anti-CD4 (RM4-5); Brilliant Violet (BV510)-conjugated anti-SiglecF (E50-2440) and anti-B220 (RA3-6B2); Brilliant Violet (BV605)-conjugated anti-CD11b (M1/70); FITC-conjugated anti-Ly6C (HK1.4) and anti-Gr1 (RB6-8C5); PE-conjugated anti-Ly6G (1A8), anti-TCRγ/δ (GL3), and anti-CD45 (30-F11); phycoerythrin-cyanine7 (PE-Cy7)-conjugated anti-CD8a (53-6.7). Antibody-stained cells were acquired with FACSAria (BD Biosciences) and analyzed with FACSDiva software Version 8.0.2 (BD Bioscience).

### Nucleic Acid Extraction and Whole-genome Sequencing

Size-matched pooled ileal tumors (containing 9–12 tumors ≥2 mm in diameter) and mouse tails were used for genomic DNA extraction (*n* = 3 mice for *Apc* or *Apc/Dok3*). Genomic DNA was extracted using lysis buffer (10 mg/L Proteinase K, 0.1 mol/L Tris-HCl pH8.0, 0.4% SDS, 5 mmol/L EDTA, 0.2 mol/L NaCl) followed by phenol-chloroform extraction and isopropanol precipitation. Extracted genomic DNA was processed at Macrogen using Truseq DNA PCR-Free library preparation (Illumina). For whole-genome sequencing (WGS), tumor DNA samples were sequenced to an average depth of 100 × coverage, and matched non-tumor (tail) DNA samples were sequenced to an average depth of 30 × coverage, with 150 bp paired-end reads on Illumina Novaseq 6000 instruments. The Illumina platform generates raw images and base calling through an integrated primary analysis software called RTA (Real-Time Analysis). The BCL/cBCL (base calls) binary was converted into FASTQ using Illumina package bcl2fastq2-v2.20.0. The demultiplexing option (–barcode-mismatches) was set to perfect match (value: 0).

### Analysis of Somatic Mutations

Candidate somatic mutations were detected through the Genomon pipeline (https://github.com/Genomon-Project/genomon-docs/tree/v2.0). GRCm38/mm10 was used as the mouse reference genome. Candidate mutations in a tumor sample were identified with the following procedure: A candidate with (i) the Fisher exact *P* ≤ 0.01; (ii) 0.1 < strand ratio < 0.9; (iii) ≥5 variant reads in the tumor sample; (iv) the variant allele frequency (VAF) in the tumor sample ≥0.02; and (v) the VAF in the matched normal sample <0.1 was adopted. ANNOVAR (https://annovar.openbioinformatics.org/en/latest/) was used to annotate the detected variants.

### Statistical Analysis

Data were analyzed by Student *t* test, Fisher exact test, or the *χ*^2^ test and were expressed as mean ± SD (Student *t* test) or percentage (Fisher exact and *χ*^2^ tests). *P* < 0.05 was considered statistically significant.

### Data Availability Statement

Sequencing FASTQ data files from WGS have been deposited in the Japanese Genotype-phenotype Archive, which is hosted by the DDBJ, under accession number DRA015135.

## Results

### Dok-3 Deficiency Leads to Malignant Progression in ApcMin/+ Mice

**FIGURE 1:**
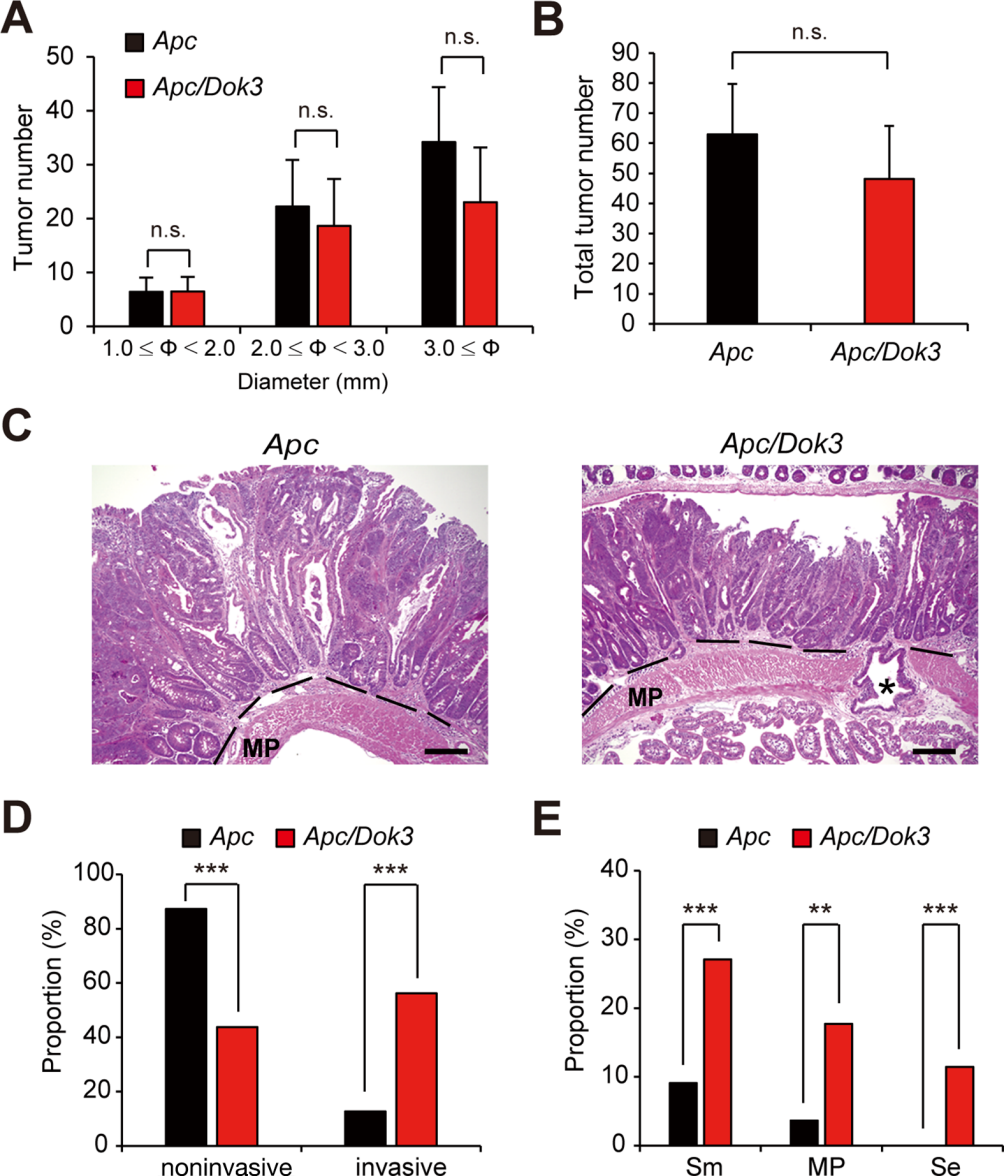
Loss of Dok-3 results in malignant progression in *Apc* mice. Tumor size distribution (**A**) and total tumor number (≥1 mm in diameter; **B**) in the small intestine at 6 months of age. All values represent the mean ± SD (*n* = 5 mice for *Apc*, *n* = 6 mice for *Apc/Dok3*). n.s., not significant by Student *t* test. **C,** H&E-stained histologic images of the tumors in the small intestines at 6 months of age. The dotted line shows the muscularis mucosae. The asterisk indicates a tumor penetrating beyond the muscularis propria (MP) and reaching the serosal surface. Scale bars, 200 μm. **D,** H&E-stained tumors ≥2 mm in diameter in the small intestines at 6–7 months of age were classified into noninvasive (tumors confined to the mucosal crypt) or invasive (tumors invading submucosa or beyond). The number of tumors classified as noninvasive or invasive is shown as a proportion of the total tumor number analyzed (110 tumors of 5 mice for *Apc*; 96 tumors of 5 mice for *Apc/Dok3*). **E,** Invasive tumors analyzed in **D** were further classified into three groups, namely tumors invading the submucosa (Sm), the muscularis propria (MP), or tumors reaching the serosal surface (Se). Invasion depth increases from Sm to Se. The number of tumors in each group is shown as a proportion of the total number of tumors (noninvasive and invasive) analyzed in **D**. **, *P* < 0.01; ***, *P* < 0.001 compared with *Apc* mice by Fisher exact test.

To investigate the role of Dok-1, -2, and -3 in the development and malignant conversion of intestinal tumors, we crossed mice lacking Dok-1 and Dok-2, the closest homologs with redundant functions among Dok family adaptors (10), or Dok-3 knockout mice with ApcMin/+ mice (abbreviated as *Apc* mice), a mouse model of intestinal tumorigenesis bearing a heterozygous mutation in the tumor suppressor gene *Apc* (13, 14). At 6 months of age, the total number of tumors (1.0 mm ≤ Φ) in the small intestines of *Apc* mice lacking Dok-1/-2 (ApcMin/+;Dok-1/-2 knockout, abbreviated as *Apc*/*Dok1*/*Dok2* mice) was significantly greater than that of *Apc* mice (Supplementary Fig. S1A). In particular, the number of relatively large (3.0 mm ≤ Φ), but not small (1.0 mm ≤ Φ < 3.0 mm), diameter tumors was significantly increased in *Apc*/*Dok1*/*Dok2* mice compared with that in *Apc* mice (Supplementary Fig. S1B), indicating that Dok-1/-2 deficiency enhances intestinal tumor growth. In contrast, neither the size nor the total number of tumors in *Apc* mice was affected by Dok-3 deficiency, comparing *Apc* and ApcMin/+;Dok-3 knockout mice (abbreviated as *Apc/Dok3* mice; Fig. 1A and B). However, *Apc/Dok3* mice, but not *Apc*/*Dok1*/*Dok2* mice, developed a significantly increased number of invasive tumors; namely those invading the submucosa, the muscularis propria, or reaching the serosal surface (abbreviated as Sm, MP, or Se in the figures, respectively; Fig. 1C–E; Supplementary Fig. S2A–S2C). Whereas most tumors in *Apc* mice were noninvasive (87%) and exhibited no deep invasion beyond the smooth muscle layer (muscularis propria), 56% of the tumors in *Apc/Dok3* mice were invasive and 11% of the tumors penetrated beyond the muscularis propria and reached the serosal surface (Fig. 1D and E). Of note, depth of invasion indicates the deepest invasion area of each invasive tumor determined by H&E-stained serial sections obtained from whole small intestines (Supplementary Fig. S3; refs. 5, 19, 20). In addition, stromal reaction associated with these tumors supports their invasiveness rather than pseudoinvasiveness (Supplementary Fig. S4; ref. 21). Also, consistent with the previous observations showing that the invasion front of human colorectal adenocarcinomas displays low proliferative activity (22), the rate of proliferating *Apc/Dok3* tumor cells in the invasion front was significantly lower than that in the tumor center (Supplementary Fig. S5A–S5C). Importantly, marked tumor invasion was also observed in colon tumors in *Apc/Dok3* mice (Supplementary Fig. S6); 90% of colorectal tumors in *Apc/Dok3* mice, but not in *Apc* mice, invaded the muscularis propria or beyond (Supplementary Fig. S6C). These results indicate that Dok-3 deficiency affects tumor malignant progression but not tumorigenesis in *Apc* mice.

### Transplantation of Dok-3–deficient Bone Marrow Cells Induces Tumor Invasion in *Apc* Mice

**FIGURE 2:**
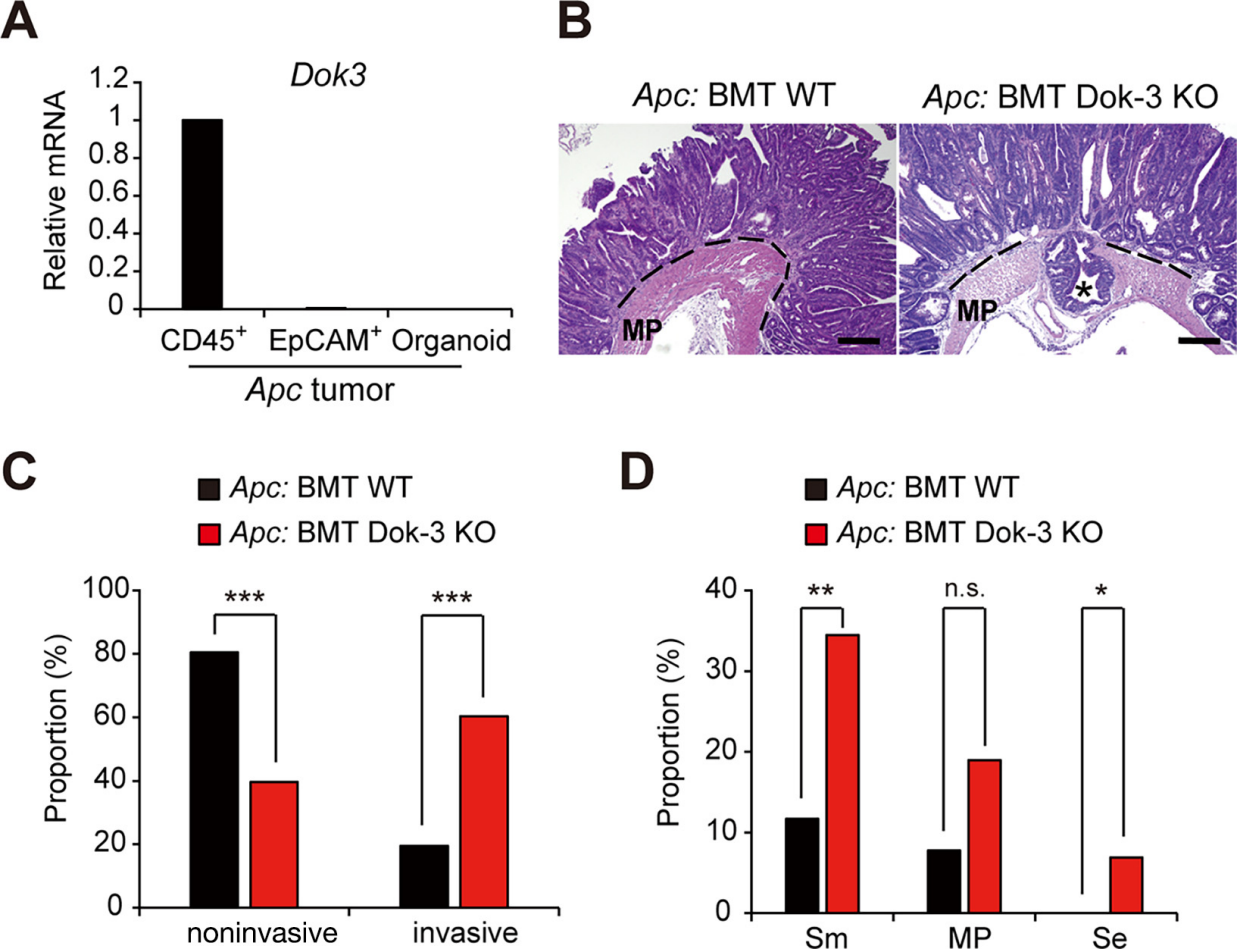
Loss of Dok-3 in bone marrow cells leads to malignant progression in *Apc* mice. **A,** The mRNA levels of *Dok3* were assessed by qRT-PCR. Leukocytes (CD45^+^) and epithelial cells (EpCAM^+^) from tumors in the small intestines of *Apc* mice at 6 months of age were fractionated by flow cytometry. Organoids were generated from tumors in the small intestines of *Apc* mice. Data were normalized against *Gapdh*. All values represent the mean ± SD (*n* = 3). **B,** H&E-stained histologic images of the tumors in the small intestines at 6 months of age. The dotted line shows the muscularis mucosae. The asterisk indicates a tumor penetrating beyond the muscularis propria (MP) and reaching the serosal surface. Scale bars, 100 μm. **C,** H&E-stained tumors ≥2 mm in diameter in the small intestines at 6–7 months of age were classified as noninvasive or invasive (as defined in Fig. 1D). The number of tumors classified as noninvasive or invasive is shown as a proportion of the total tumor number analyzed (77 tumors from 4 *Apc* mice: BMT WT; 58 tumors from 4 *Apc* mice: BMT Dok-3 KO). **D,** Invasive tumors analyzed in **C** were further classified into three groups (Sm, MP, or Se as defined in Fig. 1E) and the number of tumors in each group is shown as a proportion of the total number of tumors (noninvasive and invasive) analyzed in **C**. *, *P* < 0.05; **, *P* < 0.01; ***, *P* < 0.001 compared with *Apc* mice: BMT WT by Fisher exact test. KO, knockout; n.s., not significant.

To assess how Dok-1/-2 deficiency enhances tumor growth but Dok-3 deficiency induces malignant conversion of benign tumors, we first evaluated expression levels of the Dok family genes in tumor epithelial cells of *Apc* mice. Interestingly, although we observed mRNA expression of *Dok-1 and Dok-2* in tumor epithelial cells, we failed to detect *Dok-3* mRNA in the epithelial cells, but did find it in leukocytes (Fig. 2A; Supplementary Fig. S7). In addition, even in tumor organoid cells from *Apc* mice, the *Dok-3* mRNA expression was undetectable (Fig. 2A; Supplementary Fig. S7). Given the growth inhibitory function of Dok-1/2 (12, 17, 23), these findings suggest that the loss of Dok-1/2 enhances tumor cell growth in a tumor cell–intrinsic manner, while Dok-3 may induce malignant conversion of benign tumors in a tumor cell–extrinsic manner. Thus, we transplanted Dok-3–deficient bone marrow cells, including hematopoietic stem cells that can systemically restore blood cells, into irradiated *Apc* mice to investigate the impact of Dok-3 loss in the bone marrow–derived stromal cells on tumor invasion. Because it has been reported that irradiation required for the transplantation induces intestinal tumor invasion in *Apc* mice (24), the abdominal region containing intestines of *Apc* mice was covered with a tungsten shield to protect the region from irradiation. As expected, most tumors in *Apc* mice transplanted with bone marrow cells from WT mice (*Apc*: BMT WT) remained noninvasive (82%) and exhibited no deep invasion beyond the muscularis propria (Fig. 2B–D), similar to those observed in *Apc* mice (Fig. 1C–E). In contrast, *Apc* mice transplanted with Dok-3–deficient bone marrow cells (*Apc*: BMT Dok-3 knockout) developed invasive tumors in higher proportion (60%) than *Apc*: BMT WT, including some reaching the serosal surface (7%; Fig. 2B–D), similar in pattern to that observed in *Apc/Dok3* mice compared with *Apc* mice (Fig. 1C–E). These findings indicate that bone marrow–derived stromal cells lacking Dok-3 have the potential to induce invasion of *Apc* tumors in mice, demonstrating a previously unrecognized, tumor cell–extrinsic mechanism in malignant progression.

### Dok-3 Deficiency Induces Tumor Invasion in the Absence of Enhanced Gene Mutation in Tumors

**FIGURE 3:**
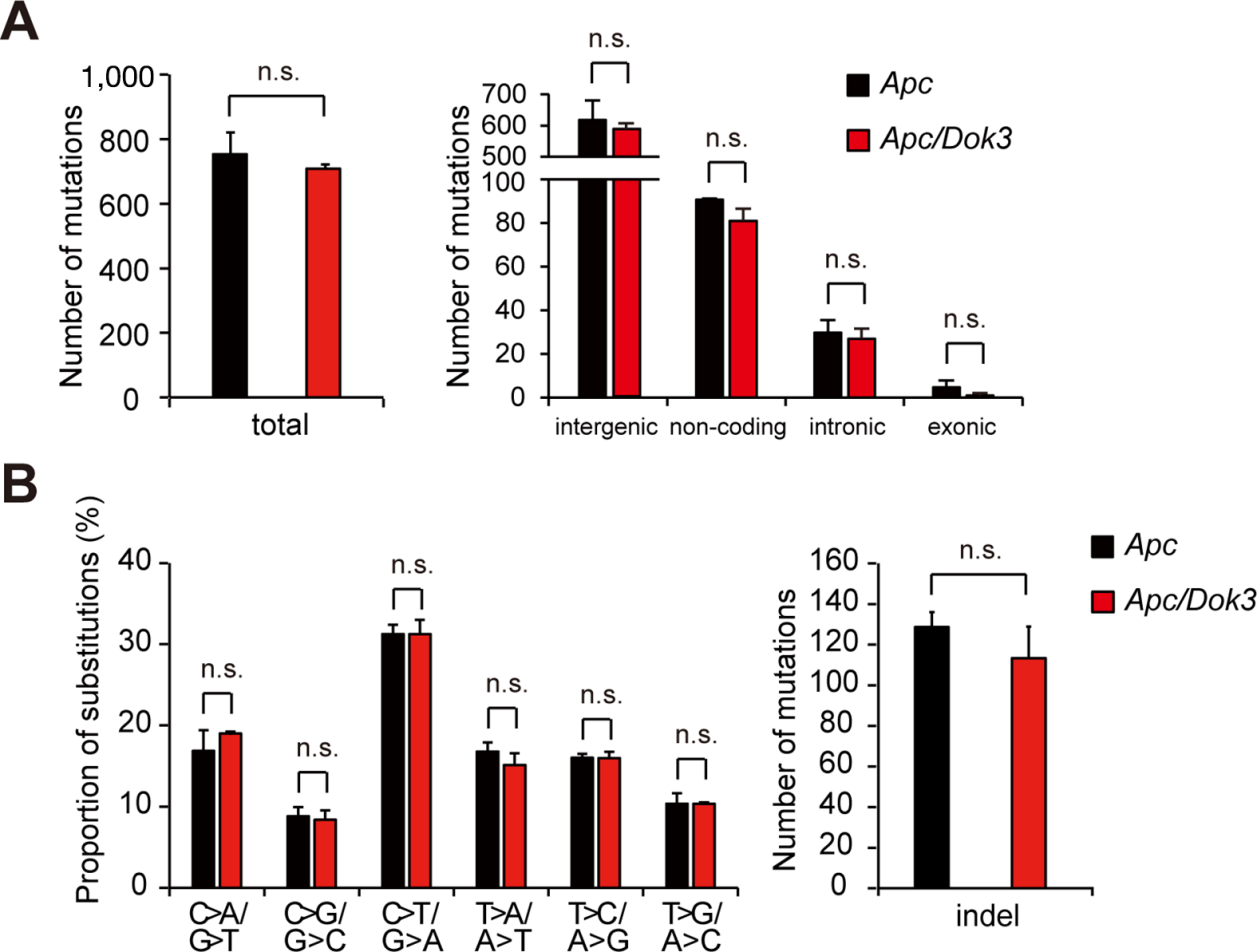
Loss of Dok-3 has no significant effect on mutation rate in tumors from *Apc* mice. Total number of mutations (**A**, Left), the number of mutations in each genomic region (**A**, Right), nucleotide substitution pattern (**B**, Left), and the number of indel mutations (**B**, Right) determined by WGS of size-matched tumors in the small intestines of *Apc* mice or *Apc/Dok3* mice at 6 months of age. All values represent the mean ± SD (*n* = 3). n.s., not significant by Student *t* test.

Accumulation of driver mutations in genes including *Apc*, *Braf, Kras*, *Smad4*, *Tp53,* or *Pik3ca* is known to cause invasive tumor development in mouse intestine (15, 25–27), and these mutations in humans are also associated with colorectal malignancies (28, 29). Moreover, benign tumors in *Apc* mutant mice acquire invasive ability due to additional driver mutations (15), and myeloid cells lacking the *Gpx4* gene induce increased mutation frequencies in tumors as reflected in their whole-exome sequences, causing malignant progression of azoxymethane (AOM)-induced colonic benign tumors (6). These findings raise the possibility that Dok-3 deficiency enhances gene mutation frequency in tumor cells bearing the *Apc*Min mutation to generate additional driver mutations required for malignant conversion. Thus, we performed WGS using tumors from *Apc* mice possessing or lacking Dok-3. The WGS data clearly demonstrate that the total number of mutations in tumors from *Apc* mice was not affected by Dok-3 deficiency (Fig. 3A). Furthermore, the distribution of mutations across the genome, their substitution patterns, and the number of insertion-deletion (indel) mutations were comparable between tumors from *Apc* mice and those from *Apc/Dok3* mice (Fig. 3A and B). Indeed, somatic mutations in 95 genes significantly mutated in colorectal cancer, including six known driver genes (*APC*, *BRAF*, *KRAS*, *SMAD4, TP53,* and *PIK3CA*; refs. 28, 29; Supplementary Table S1), were not observed in tumors from *Apc* mice, irrespective of the presence or absence of Dok-3. These findings together indicate that Dok-3 deficiency that drives malignant conversion of benign tumors had no apparent effect on mutagenesis in tumors, suggesting a mutagenesis-independent progression of malignancy induced by non-tumor cells.

### T, but not B, Lymphocytes are Required for Malignant Conversion of Benign Tumors in *Apc/Dok3* Mice

**FIGURE 4:**
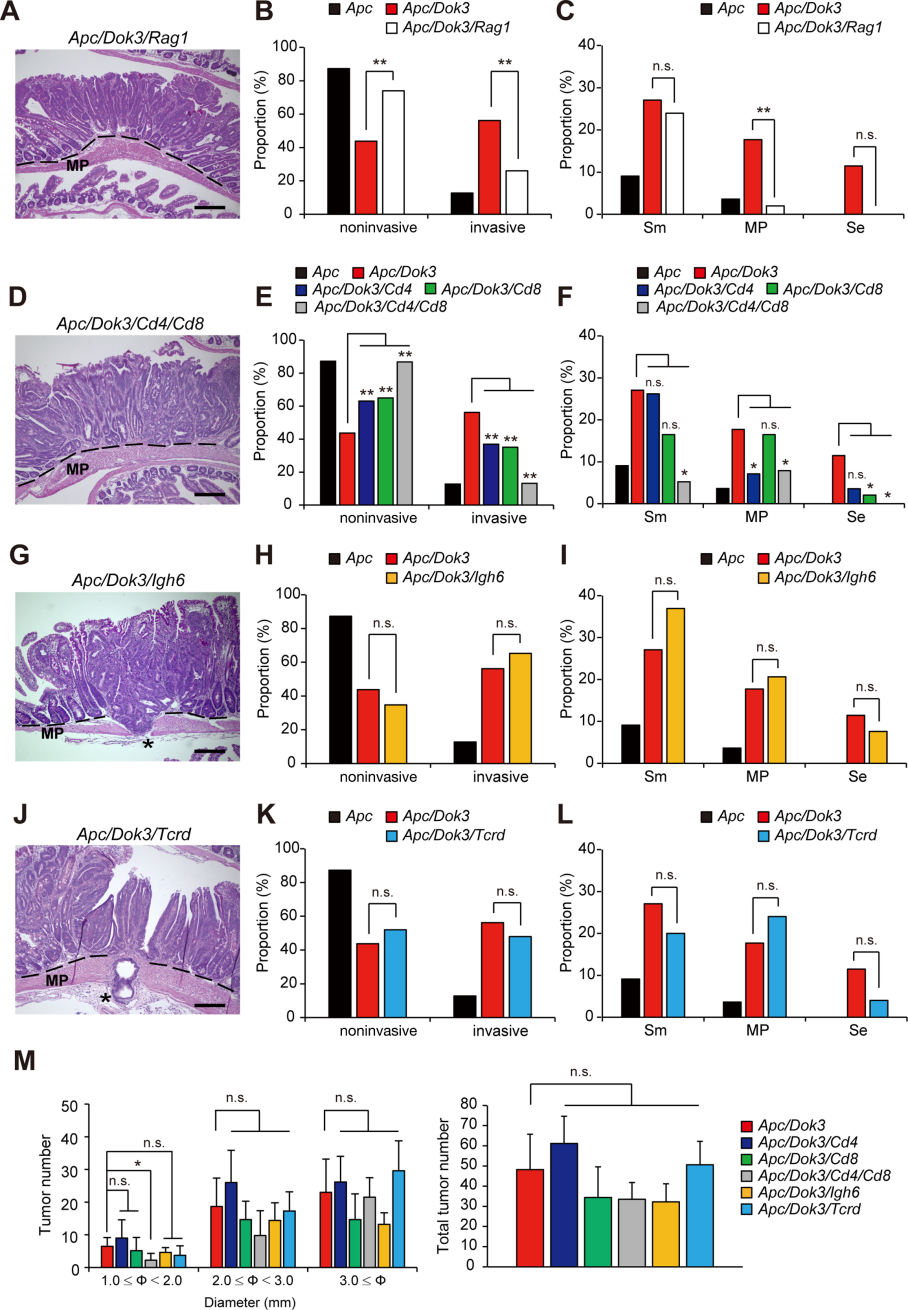
CD4^+^ and CD8^+^ T cells are essential for tumor invasion in *Apc/Dok3* mice. H&E-stained histologic images of the tumors in the small intestines of *Apc/Dok3* mice lacking Rag1 (*Apc/Dok3/Rag1*; **A**), *Apc/Dok3* mice lacking Cd4 and Cd8 (*Apc/Dok3/Cd4/Cd8*; **D**), *Apc/Dok3* mice lacking Igh6 (*Apc/Dok3/Igh6*; **G**), or *Apc/Dok3* mice lacking Tcrd (*Apc/Dok3/Tcrd*; **J**) at 6–7 months of age. The dotted line indicates the muscularis mucosae. Asterisks indicate tumors penetrating beyond the muscularis propria (MP) and reaching the serosal surface. Scale bars, 200 μm. **B**, **E**, **H**, and **K**, H&E-stained tumors ≥2 mm in diameter in the small intestines at 6–7 months of age were classified into noninvasive or invasive (as defined in Fig. 1D). The numbers of tumors classified as noninvasive or invasive are shown as proportions of total tumor numbers analyzed in each case (110 tumors from 5 *Apc* mice; 96 tumors from 5 *Apc/Dok3* mice; 48 tumors from 5 *Apc/Dok3/Rag1* mice; 84 tumors from 3 *Apc/Dok3/Cd4* mice; 97 tumors from 3 *Apc/Dok3/Cd8* mice; 76 tumors from 3 *Apc/Dok3/Cd4/Cd8 mice*; 92 tumors from 4 *Apc/Dok3/Igh6 mice*; 50 tumors from 3 *Apc/Dok3/Tcrd* mice). **C**, **F**, **I**, and **L**, Invasive tumors analyzed in **B**, **E**, **H**, and **K** were further classified into three groups (Sm, MP, or Se as defined in Fig. 1E) and the numbers of tumors in each group are shown as proportions of the total number of tumors (noninvasive and invasive) analyzed in **B**, **E**, **H**, and **K**, respectively. *, *P* < 0.05; **, *P* < 0.01 compared with *Apc/Dok3* mice by Fisher exact test (comparisons between *Apc* and *Apc/Dok3* mice are not included). n.s., not significant. **M,** Tumor size distribution (left) and total tumor number (≥1 mm in diameter; right) in the small intestine at 6 months of age. All values represent the mean ± SD (*n* = 6 mice for *Apc/Dok3*, *n* = 6 mice for *Apc/Dok3*/Cd4, *n* = 8 mice for *Apc/Dok3/Cd8*, *n* = 4 mice for *Apc/Dok3/Cd4/Cd8*, *n* = 5 mice for *Apc/Dok3/Igh6, n* = 7 mice for *Apc/Dok3/Tcrd*). *, *P* < 0.05 compared with *Apc/Dok3* mice by Student *t* test. n.s., not significant.

Because hematopoietic reconstitution of *Apc* mice with Dok-3 knockout bone marrow cells induced a level of invasive tumors comparable with *Apc/Dok3* mice (Figs. 1C–E and 2B–D), we first examined the impact of lymphocyte depletion in *Apc/Dok3* mice by crossing those mice with DNA recombinase Rag1-deficient mice, which lack mature T and B lymphocytes (30). Interestingly, the rate of invasive tumor occurrence in *Apc/Dok3* mice lacking Rag1 (26%) was significantly lower than that in *Apc/Dok3* mice (56%; Fig. 4A and B). Furthermore, the rate of tumors invading the muscularis propria or beyond in *Apc/Dok3* mice, 18% or 11%, was reduced to 2% or 0%, respectively, in *Apc/Dok3* mice lacking Rag1 (Fig. 4C). Therefore, to identify lymphocyte populations essential for tumor invasion in *Apc/Dok3* mice, we crossed *Apc/Dok3* mice with CD4 and/or CD8-deficient mice to deplete CD4^+^ and/or CD8^+^ T cells. Note that the development of B cells and myeloid cells is unaltered by CD4 and CD8 deficiency in mice (31, 32). The rate of invasive tumors in *Apc/Dok3* mice lacking CD4^+^ T cells (37%) or CD8^+^ T cells (35%) or CD4^+^ and CD8^+^ T cells (13%) was significantly lower than that in *Apc/Dok3* mice (56%; Fig. 4D and E), and the rate of invasive tumors in *Apc/Dok3* mice lacking both CD4^+^ and CD8^+^ T cells was indistinguishable to that in *Apc* mice (13% vs. 13%; Fig. 4E). Furthermore, 11% of the tumors reached the serosal surface in *Apc/Dok3* mice, whereas such invasive tumors were not observed in either *Apc/Dok3* mice lacking CD4/CD8 or *Apc* mice (Fig. 4F). In contrast, *Apc/Dok3* mice lacking Igh6 or Tcrd, which lack B lymphocytes or γδT cells, respectively, show similar levels of malignant progression to *Apc/Dok3* mice (Fig. 4G–L). In addition, aside from a suppression of small tumor (1.0 mm ≤ Φ < 2.0 mm) numbers observed in *Apc/Dok3/Cd4/Cd8* mice compared with *Apc/Dok3* mice, tumorigenesis in *Apc/Dok3* mice was not significantly affected by depletion of any of the tested lymphocyte populations (Fig. 4M). Together, these findings demonstrate that CD4^+^ and CD8^+^ T cells, but not B and γδT cells, have an essential role in malignant progression of benign tumors in *Apc/Dok3* mice, although the underlying mechanisms are yet unclear.

### Dok-3 Deficiency has no Apparent Effect on Inflammatory Status of Tumors

**FIGURE 5:**
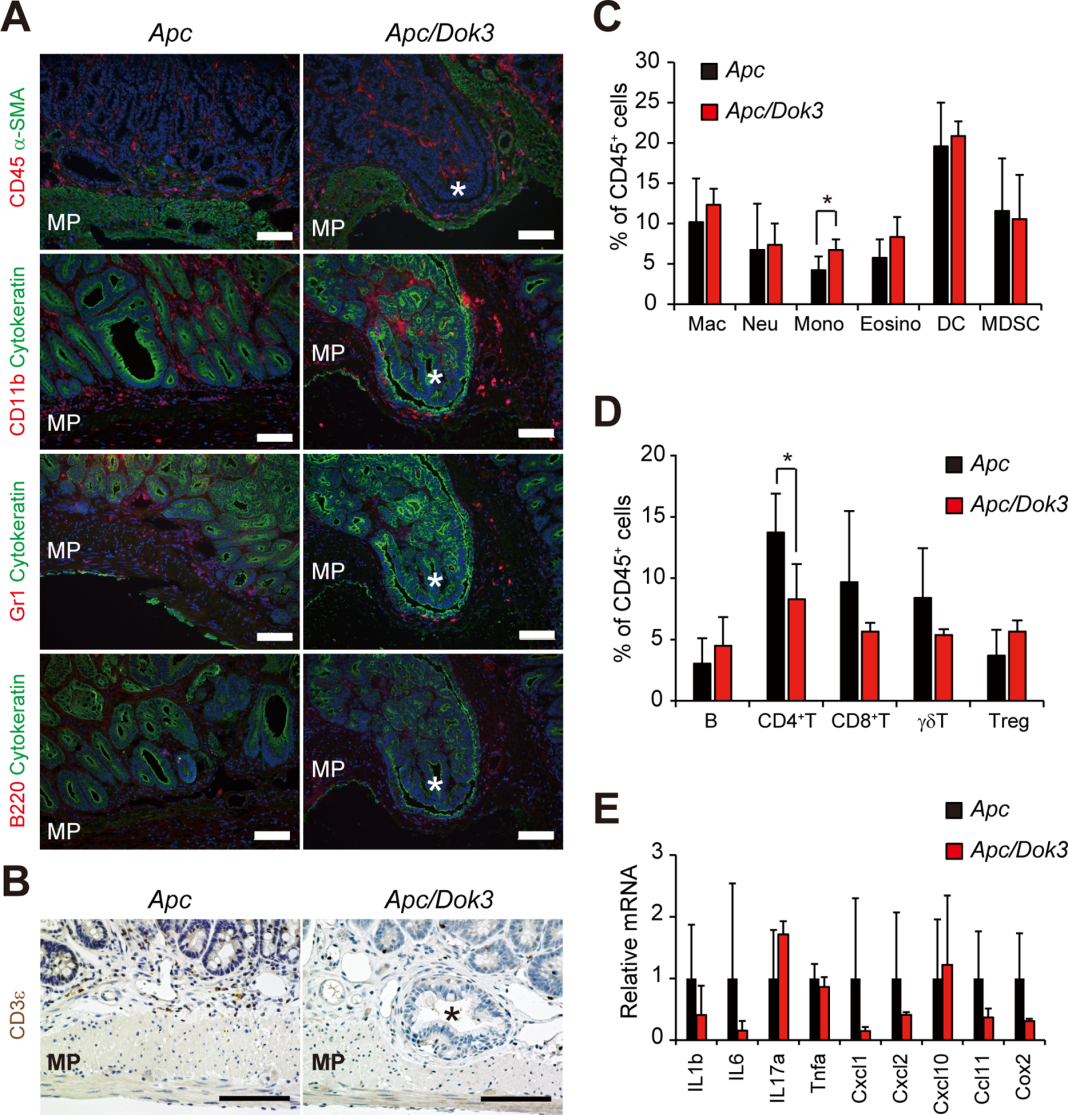
Inflammatory response is not elevated in tumors of *Apc/Dok3* mice. **A** and **B,** Tumors in the small intestines of *Apc* mice and *Apc/Dok3* mice at 6 months of age were stained for the cell surface markers shown. α-SMA, a smooth muscle cell marker; Cytokeratin, an epithelial cell marker; CD45, a pan-leukocyte marker; CD11b, a myeloid cell marker, Gr1, a granulocyte marker; B220, a pan-B cell marker; CD3ε, a pan-T cell marker. Asterisks show the tumor invading the muscularis propria (MP). Scale bars, 100 μm. **C** and **D,** Flow cytometric analysis of different immune cell populations in CD45^+^ cells in tumors in the small intestines of *Apc* mice or *Apc/Dok3* mice at 6 months of age. All values represent the mean ± SD (*n* = 6). No significant difference in frequencies of individual immune cell populations was observed between tumors of *Apc* mice and those of *Apc/Dok3* mice by Student *t* test aside from monocyte (**C**) and CD4^+^ T-cell (**D**) populations. *, *P* < 0.05 compared with *Apc* mice by Student *t* test. Mac, macrophage. Neu, neutrophil. Mono, monocyte. Eosino, eosinophil. DC, dendritic cell, MDSC, myeloid-derived suppressor cell. **E,** The mRNA levels of inflammatory markers in size-matched tumors in the small intestines of *Apc/Dok3* mice relative to the mean levels in tumors of *Apc* mice. Data were normalized against *Gapdh*. All values represent the mean ± SD (*n* = 3). No significant difference was observed between tumors of *Apc* mice and those of *Apc/Dok3* mice by Student *t* test.

Inflammatory responses play critical roles in the development and malignant progression of tumors. Given that immune cells infiltrating the microenvironment, a hallmark of inflammation, is known to affect tumor cell malignancy (33), we examined the impact of Dok-3 deficiency in *Apc* mice on inflammatory status of tumors. Although CD4^+^ and CD8^+^ T cells are essential for malignant progression in *Apc/Dok3* mice (Fig. 4), leukocytes, including these T cells, myeloid cells, granulocytes, and B cells did not markedly accumulate in tumors of *Apc/Dok3* mice beyond the levels observed in *Apc* mice (Fig 5A and B). In addition, flow cytometric analysis showed that the frequencies of individual immune cell populations among CD45^+^ leukocytes in tumors of *Apc/Dok3* mice were similar to those in *Apc* mice aside from monocyte and CD4^+^ T-cell populations, which were slightly higher and lower in *Apc* tumors lacking Dok-3, respectively (Fig. 5C and D). Furthermore, expression levels of inflammatory cytokines, chemokines and COX-2 in tumors of *Apc/Dok3* mice were not significantly increased in comparison with those observed in tumors of *Apc* mice (Fig. 5E). These findings demonstrate that generation of an inflammatory microenvironment is unlikely to be a primary driver of malignant progression in *Apc/Dok3* mice.

## Discussion

In the current study, we show that Dok-3 deficiency induces malignant progression in the intestinal benign tumor model, *Apc* mice (Fig. 1C–E). Interestingly, the loss of Dok-1/-2, the homologs of Dok-3, did not significantly affect malignant progression but rather enhanced tumor growth in *Apc* mice (Supplementary Fig. S1 and S2), indicating distinct roles of Dok-3 and Dok-1/-2. Although the underlying mechanism remains unclear, distinct binding partners of Dok-1/-2 and Dok-3 adaptors might cause these differences. For instance, p120 rasGAP selectively binds Dok-1/2 (10); in contrast, Grb2 preferentially binds Dok-3 (34), though any involvement of Grb2 in tumor invasion remains unclear.

Although we cannot completely exclude the possibility that the loss of Dok-3 in intestinal epithelial cells by itself can induce malignant progression, the expression of *Dok3* mRNA was not detectable in either quantitative or semiquantitative RT-PCR analyses (Fig. 2A; Supplementary Fig. S7). Therefore, it is unlikely that the loss of residual Dok-3 expression causes malignant progression in *Apc* mice. Indeed, transplantation of Dok-3–deficient bone marrow cells induced tumor invasion in *Apc* mice (Fig. 2B–D) to an extent similar to that observed in *Apc/Dok3* mice (Fig. 1D and E), indicating that Dok-3–deficient non-tumor cells have the potential to induce malignant conversion of benign tumors, namely a previously unknown, stromal cell–induced malignant progression in a spontaneous tumor model. Although the underlying mechanisms of Dok-3 loss–induced malignant conversion await further investigation, our examination of *Apc/Dok3* invasive tumors found no signs of epithelial–mesenchymal transition (EMT), which is involved in a wide range of malignant progression (35). In detail, Dok-3 loss had no impact on the expression of EMT markers in *Apc* tumors, including EMT-inducing transcription factors (Supplementary Fig. S5E). Furthermore, tumor cells in the center and invasion front of tumors retained expression of epithelial cell marker E-Cadherin in *Apc/Dok3* mice (Supplementary Fig. S5A–S5D), whose loss is a hallmark of EMT. Tumor invasion without inducing EMT is also reported in *Apc* mutant mice lacking the negative regulator of Notch signaling, Aes, in tumor epithelium (19). However, the impact of Dok-3 loss on Notch signaling remains to be investigated.

As mentioned above, the loss of Gpx4 in myeloid cells induced malignant progression in the AOM-induced colonic benign tumor model (6). However, the rate of invasive colonic tumors in myeloid Gpx4-deficient AOM mice (∼10%) was about 10-fold lower than that in *Apc/Dok3* mice (100%; Supplementary Fig. S6B). Furthermore, although Gpx4 deficiency in myeloid cells significantly increased exome mutagenesis in tumors (6), which eventually led to malignant progression, our WGS analysis demonstrates that the loss of Dok-3 did not enhance genomic mutations in tumors of *Apc* mice, in terms of both levels and patterns of mutations (Fig. 3). Thus, these findings suggest a novel tumor cell mutagenesis-independent mechanism underlying malignant progression induced by the loss of Dok-3 in stromal cells.

Interestingly, depletion of CD4^+^ or CD8^+^ T cells partially but significantly inhibited the tumor invasion observed in *Apc/Dok3* mice (Fig. 4D–F). In addition, *Apc/Dok3* mice lacking both CD4^+^ and CD8^+^ T cells showed no enhancement of malignant progression in comparison with *Apc* mice (13% vs. 13%: the rate of invasive tumors in Fig. 4E), indicating an essential and cooperative role of CD4^+^ and CD8^+^ T cells in the Dok-3 loss–induced tumor invasion in *Apc* mice. In contrast, depletion of B or γδT lymphocytes had no impact on tumor invasion in *Apc/Dok3* mice (Fig. 4G–L). However, the mechanism by which CD4^+^ and CD8^+^ T cells mediate malignant progression in *Apc/Dok3* mice has not yet been elucidated. Given that tumors, including invasion fronts, in *Apc/Dok3* mice did not show any increased infiltration of CD4^+^ and CD8^+^ T cells in comparison with *Apc* mice (Fig. 5A, B, and D), these lymphocytes might exert distal effects, for instance, via exosomes, because immune cell–derived exosomes are known to regulate cancer progression and metastasis (36). Indeed, CD8^+^ T cell–derived exosomes are reported to promote invasion of melanoma and lung cancer cells by increasing the expression of MMP9 via Fas signaling in the malignant cells (37). Furthermore, given the possible distal effects of T cells, comparison between Dok-3 expression in peripheral CD4^+^ and CD8^+^ T cells from adenocarcinoma-bearing patients and that from non–adenocarcinoma-bearing patients with familial adenomatous polyposis might help to gain insight into the clinical relevance of Dok-3 loss–induced malignant progression, even though such datasets appear unavailable at this point.

It has been shown that Th2-type CD4^+^ T cells indirectly promote invasion and subsequent metastasis of malignant tumors (mammary adenocarcinomas) by directly regulating tumor-associated macrophages (38), which are the most abundant immune cells in the TME and promote cancer initiation and malignant progression (39). Although it is unknown whether macrophages are involved in malignant conversion of benign tumors in ApcMin/+ mice lacking Dok-3, it might be possible that Dok-3–deficient macrophages play a role in the malignant progression by cooperating with CD4^+^ and CD8^+^ T cells. However, we could not investigate the involvement of macrophages in tumor invasion in *Apc/Dok3* mice, because we failed in our attempt to breed mice lacking Dok-3 and Csf1, the latter of which is critical for development of macrophages (40). Namely, when crossing between ApcMin/+;Dok-3 knockout mice bearing a heterozygous mutation for *Csf1* (*Csf1^op^*/+; ref. 41) and Dok-3 knockout;*Csf1^op^*/+ mice, *Csf1^op^* homozygous mutant mice (*Csf1*^op^/^op^) lacking Dok-3 were absent in the expected numbers at weaning regardless of *Apc* mutation status (Supplementary Table S2) save for one ApcMin/+;Dok-3 knockout mouse, which died at 1 month of age. These results suggest severe lethality of Dok-3 knockout;*Csf1*^op^/^op^ mice for reasons not understood.

Tumor-intrinsic accumulation of genetic alterations is reported to drive malignant progression by shaping the inflammatory microenvironment. For instance, the loss of Smad4 in tumor epithelial cells mutated in *Apc* enhances expression of the inflammatory chemokine CCL9 to promote malignant progression of the *Apc* mutant tumors by recruiting MMP-expressing CCR1^+^ bone marrow–derived cells as mentioned earlier (5). However, enhanced gene mutations or inflammatory responses in tumors were not detected in *Apc/Dok3* mice (Figs. 3 and 5), suggesting an as yet unidentified mechanism underlying the malignant progression.

Together, our findings uncover previously unrecognized tumor cell–extrinsic cues that can induce malignant conversion of benign tumors irrespective of exacerbating gene mutations or inflammatory responses in tumors. However, the current study does not sufficiently address mechanisms, including how T cells and potentially other stromal cells promote tumor invasion, nor the clinical relevance of the proposed concept. Thus, further studies are required for better understanding stromal cell–driven malignant progression and developing new therapeutic strategies targeting such malignant progression.

## Supplementary Material

Figure S1 Loss of Dok-1/-2 enhances intestinal tumor growth in Apc mice.

Table S1 95 genes significantly mutated in human colorectal cancer.

Figure S2 Loss of Dok-1/-2 has no significant effect on invasiveness of tumors in Apc mice.

Table S2 Dok-3 KO mice bearing Csf1 homozygous mutations are not observed at the expected Mendelian ratios at weaning.

Figure S3 Assessment of invasion depth.

Figure S4 Stromal reaction associated with invasive tumors of Apc/Dok3 mice.

Figure S5 Characterization of invasive tumors in Apc/Dok3 mice.

Figure S6 Dok-3 deficiency causes malignant progression of colorectal tumors of Apc mice.

Figure S7 mRNA expression of Dok-1 and Dok-2, but not Dok-3, is detectable in tumor epithelial cells.
