# Supplementary material for: Loss of Dok-3 in Non-tumor Cells Induces Malignant Transformation of Benign Epithelial Tumor Cells of the Intestine
Source: Cancer Res Commun. 2022 Dec 8;2(12):1590–600. doi: 10.1158/2767-9764.CRC-22-0347 (PMC10035524; doi:10.1158/2767-9764.CRC-22-0347)
Supplement: Figure S1 — Loss of Dok-1/-2 enhances intestinal tumor growth in Apc mice. [file crc-22-0347-s01.pdf]

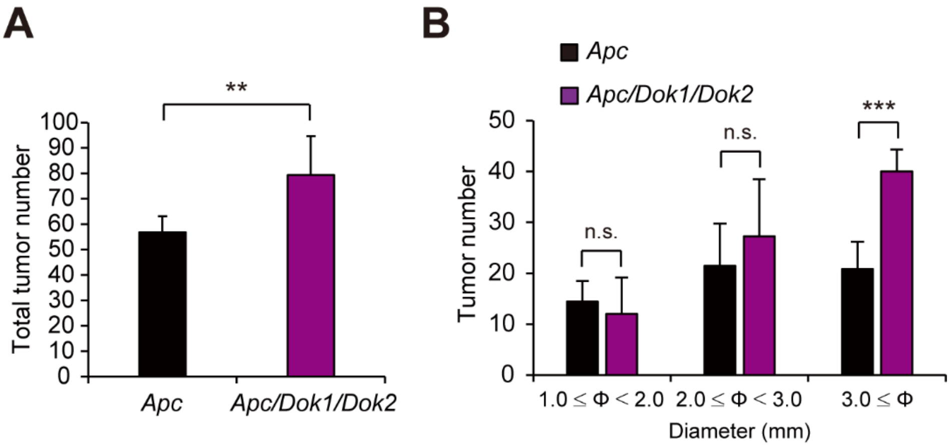

Supplementary Figure S1. Loss of Dok-1/-2 enhances intestinal tumor growth in *Apc* mice. (A, B) Total tumor number (≥ 1 mm in diameter) (A) and tumor size distribution (B) in the small intestine at 6 months of age. All values represent the mean ± SD (n = 5 mice for *Apc*, n = 7 mice for *Apc/Dok1/Dok2*). **$P < 0.01$; ***$P < 0.001$ compared with *Apc* mice by Student's *t*-test. n.s., not significant.
