# Supplementary material for: Loss of Dok-3 in Non-tumor Cells Induces Malignant Transformation of Benign Epithelial Tumor Cells of the Intestine
Source: Cancer Res Commun. 2022 Dec 8;2(12):1590–600. doi: 10.1158/2767-9764.CRC-22-0347 (PMC10035524; doi:10.1158/2767-9764.CRC-22-0347)
Supplement: Table S1 — 95 genes significantly mutated in human colorectal cancer. [file crc-22-0347-s02.pdf]

ABCF2	ACOXL	ACVR1B	ACVR2A	ADAMTS3
ADD2	AHI1	APC	ARID1A	ARPC1B
ASXL1	AXIN2	BCL9L	BRAF	C6orf136
C7orf31	CDH4	CTCF	CTNNB1	DAO
DGKA	DIAPH1	DRD3	DUSP16	EDNRB
EI24	ELF3	FAM123B	FAM171B	FBXW7
FLYWCH1	GDF5	GORASP1	GRHPR	HEATR2
HSPA1L	HTR3C	IL7R	ING1	ITIH1
KIAA1804	KLF5	KRAS	LIMK1	MAP2K1
MAP2K7	MARK2	MGAT3	MOV10	MST4
MUC17	MYBL2	NAT10	NCAPD3	NEK2
NKTR	NRAS	OCRL	PABPC1L	PACSIN1
PAN3	PCBP1	PIK3CA	PRKCQ	PTEN
RANBP9	RB1	RBM10	RBM12	RNF43
RUFY1	SAMM50	SERPING1	SIN3A	SMAD2
SMAD4	SOAT1	SOX9	SRRT	SSH1
SYNCRIP	SYNGR2	TCF7	TCF7L2	TDRD1
TEX14	TGFBR2	TGIF1	TMEM201	TPTE2
TRP53	UBR1	USP5	WDR86	ZFP36L2

Supplementary Table S1. 95 genes significantly mutated in human colorectal cancer. 95 genes were identified as genes recurrently mutated to a statistically significant extent in large-scale sequencing studies of human colorectal cancer (26, 27).
