# Supplementary material for: Loss of Dok-3 in Non-tumor Cells Induces Malignant Transformation of Benign Epithelial Tumor Cells of the Intestine
Source: Cancer Res Commun. 2022 Dec 8;2(12):1590–600. doi: 10.1158/2767-9764.CRC-22-0347 (PMC10035524; doi:10.1158/2767-9764.CRC-22-0347)
Supplement: Figure S2 — Loss of Dok-1/-2 has no significant effect on invasiveness of tumors in Apc mice. [file crc-22-0347-s03.pdf]

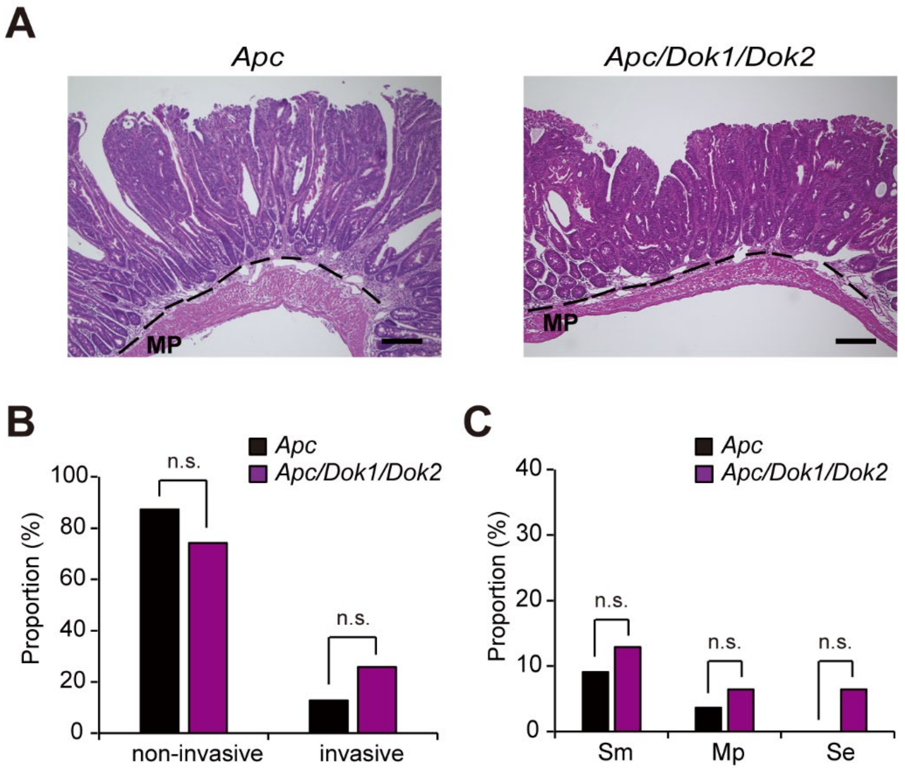


**Supplementary Figure S2. Loss of Dok-1/-2 has no significant effect on invasiveness of tumors in *Apc* mice.** (A) H&E-stained histological images of the tumors in the small intestines at 6 months of age. The dotted line shows the muscularis mucosae. MP, muscularis propria. Scale bars, 200μm. (B) H&E-stained tumors ≥ 2 mm in diameter in the small intestines at 6-7 months of age were classified as non-invasive or invasive (as defined in Fig. 1D). The number of tumors classified as non-invasive or invasive is shown as a proportion of the total tumor number analyzed (110 tumors from 5 *Apc* mice; 39 tumors from 2 *Apc/Dok1/Dok2* mice). (C) Invasive tumors analyzed in B were further classified into 3 groups (Sm, MP, or Se as defined in Fig. 1E) and the number of tumors in each group is shown as a proportion of the total number of tumors (non-invasive and invasive) analyzed in B. n.s., not significant by Fisher's exact test.
