# Supplementary material for: Loss of Dok-3 in Non-tumor Cells Induces Malignant Transformation of Benign Epithelial Tumor Cells of the Intestine
Source: Cancer Res Commun. 2022 Dec 8;2(12):1590–600. doi: 10.1158/2767-9764.CRC-22-0347 (PMC10035524; doi:10.1158/2767-9764.CRC-22-0347)
Supplement: Table S2 — Dok-3 KO mice bearing Csf1 homozygous mutations are not observed at the expected Mendelian ratios at weaning. [file crc-22-0347-s04.pdf]

Genotype	Expected number (%)	Observed number (%)
Dok3$^{-/-}$;Csf1$^{+/+}$	7 (12.5%)	13 (23.2%)
Dok3$^{-/-}$;Csf1$^{op/+}$	14 (25%)	18 (32.1%)
Dok3$^{-/-}$;Csf1$^{op/op}$	7 (12.5%)	0 (0%)
ApcMin/+ ;Dok3$^{-/-}$;Csf1$^{+/+}$	7 (12.5%)	14 (25%)
ApcMin/+; Dok3$^{-/-}$;Csf1$^{op/+}$	14 (25%)	10 (17.9%)
ApcMin/+ ;Dok3$^{-/-}$;Csf1$^{op/op}$	7 (12.5%)	1 (1.8%)

Supplementary Table S2. Dok-3 KO mice bearing *Csf1* homozygous mutations are not observed at the expected Mendelian ratios at weaning. *Apc/Dok3* mice heterozygous for *Csf1*^{op} (ApcMin/+;Dok-3 KO;Csp1$^{op/+}$) were crossed with Dok-3 KO mice heterozygous for *Csf1*^{op} (Dok-3 KO;Csp1$^{op/+}$) and the resulting progeny were genotyped at approximately 2 weeks of age. Expected genotypes with their expected numbers vs observed numbers of progeny are shown. A chi-square test was used to determine that the difference between the expected and observed frequency distribution is statistically significant, with $P < 0.001$.
