# Supplementary material for: Loss of Dok-3 in Non-tumor Cells Induces Malignant Transformation of Benign Epithelial Tumor Cells of the Intestine
Source: Cancer Res Commun. 2022 Dec 8;2(12):1590–600. doi: 10.1158/2767-9764.CRC-22-0347 (PMC10035524; doi:10.1158/2767-9764.CRC-22-0347)
Supplement: Figure S3 — Assessment of invasion depth. [file crc-22-0347-s05.pdf]

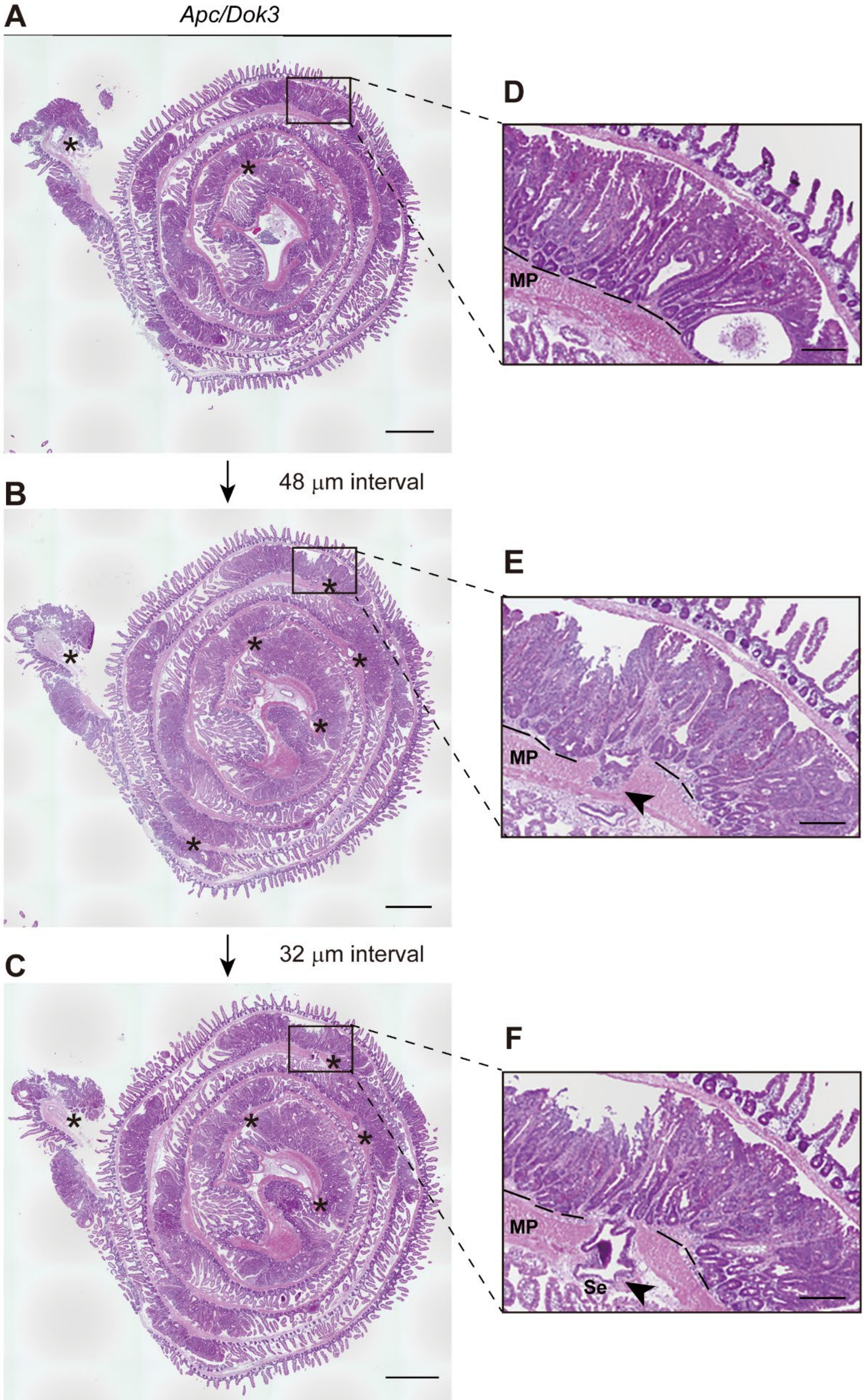

A
Apc/Dok3
B
48 μm interval
C
32 μm interval
D
MP
E
MP
F
MP
Se

Supplementary Figure S3. Assessment of invasion depth. Serial sections of whole small intestines of *Apc/Dok3* mice were prepared at 4 μm thickness and stained with H&E at approximately 16 μm intervals. (A-C) H&E-stained images of small intestinal "Swiss-roll" sections captured at different depths. The depth of each section increases from A to C. Asterisks show tumors invading the submucosa or beyond. Scale bars, 1 mm. (D, E, and F: higher magnification of the boxed area of A, B, and C, respectively) Different cross-sectional images of the same tumor. No invasion is observed in D, but with increasing section depth, muscularis propria invasion (arrowhead in E) or subserosal invasion reaching the serosal surface (arrowhead in F) is observed. Accordingly, the tumor is classified as Se. Dotted line, muscularis mucosae. MP, muscularis propria. Se, serosa. Scale bars, 200 μm.
