# Supplementary material for: Loss of Dok-3 in Non-tumor Cells Induces Malignant Transformation of Benign Epithelial Tumor Cells of the Intestine
Source: Cancer Res Commun. 2022 Dec 8;2(12):1590–600. doi: 10.1158/2767-9764.CRC-22-0347 (PMC10035524; doi:10.1158/2767-9764.CRC-22-0347)
Supplement: Figure S4 — Stromal reaction associated with invasive tumors of Apc/Dok3 mice. [file crc-22-0347-s06.pdf]

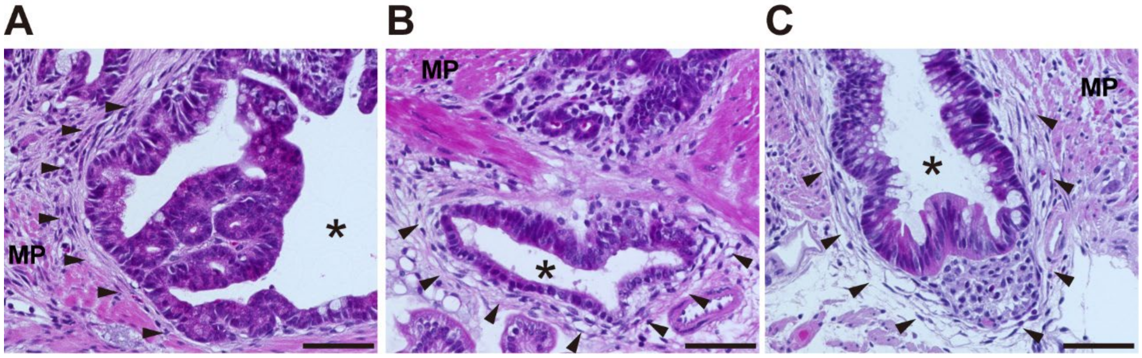

Supplementary Figure S4. Stromal reaction associated with invasive tumors of *Apc/Dok3* mice.

H&E-stained histological images of the tumors in the small intestine (A, B) or the colon (C) at 6-7 months of age. The asterisks indicate a tumor invading the muscularis propria (MP) (A) or tumors reaching the serosal surface (B, C). The arrowheads indicate the stromal reaction at the invasion front. Scale bars, 100 μm.
