# Supplementary material for: Loss of Dok-3 in Non-tumor Cells Induces Malignant Transformation of Benign Epithelial Tumor Cells of the Intestine
Source: Cancer Res Commun. 2022 Dec 8;2(12):1590–600. doi: 10.1158/2767-9764.CRC-22-0347 (PMC10035524; doi:10.1158/2767-9764.CRC-22-0347)
Supplement: Figure S5 — Characterization of invasive tumors in Apc/Dok3 mice. [file crc-22-0347-s07.pdf]

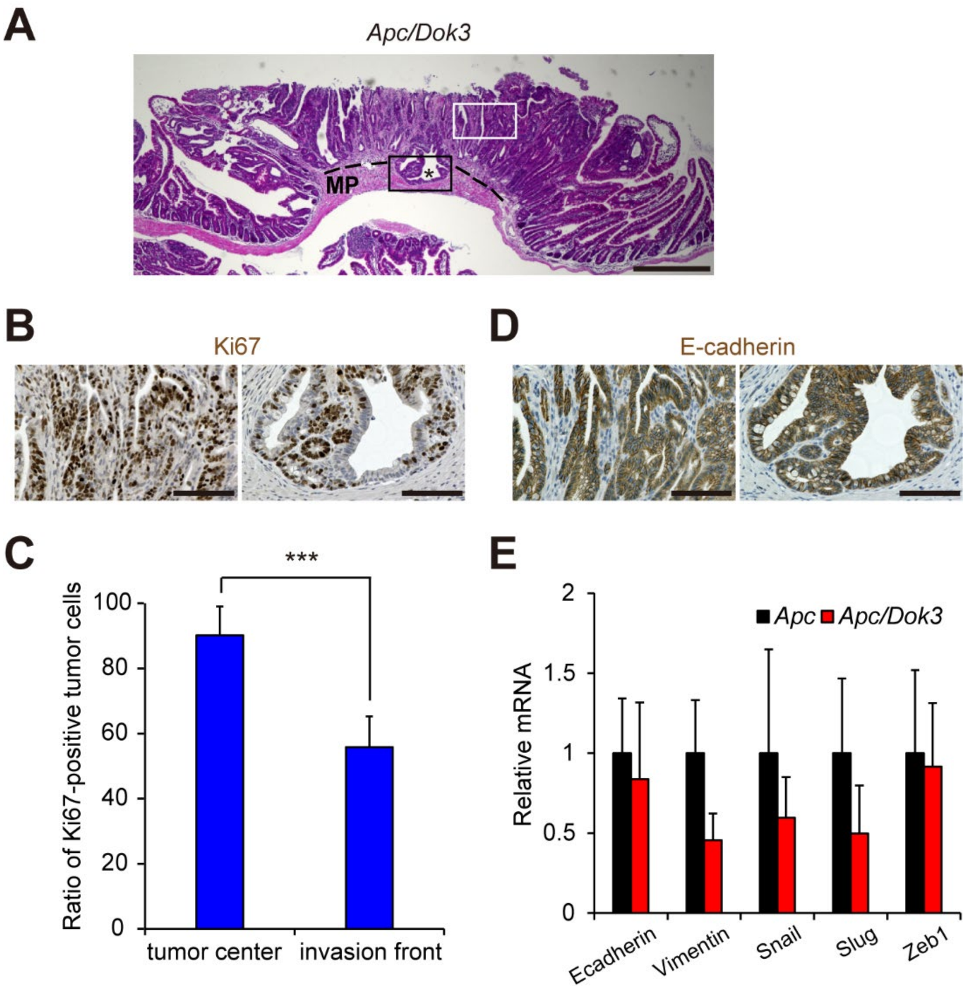

Supplementary Figure S5. Characterization of invasive tumors in *Apc/Dok3* mice

(A) A H&E-stained histological image of the tumor in the small intestine at 6 months of age. The dotted line shows the muscularis mucosae. The asterisk shows the tumor invading the muscularis propria (MP). Scale bar, 500 µm. (B, D) Higher magnification of the boxed areas in (A) representing tumor center (Left) and invasion front (Right) of the tumor stained for proliferation marker Ki67 (B) and epithelial cell marker E-Cadherin (D). Scale bars, 100 µm. (C) Percentages of Ki67-positive tumor cells in tumor center and invasion front of tumors in the small intestines of *Apc/Dok3* mice at 6 months of age. All values represent the mean ± SD (n = 15). ***$P < 0.001$ compared with tumor center by Student's t-test. (E) The mRNA levels of EMT markers in size-matched tumors in the small intestines of *Apc/Dok3* mice relative to the mean levels in tumors of *Apc* mice. Ecadherin, an epithelial cell marker; Vimentin, a mesenchymal cell marker; Snail, Slug, and Zeb1, EMT-inducing transcription factors. Data were normalized against *Gapdh*. All values represent the mean ± SD (n = 3). No significant difference was observed between

tumors of *Apc* mice and those of *Apc/Dok3* mice by Student's *t*-test.
