# Supplementary material for: Loss of Dok-3 in Non-tumor Cells Induces Malignant Transformation of Benign Epithelial Tumor Cells of the Intestine
Source: Cancer Res Commun. 2022 Dec 8;2(12):1590–600. doi: 10.1158/2767-9764.CRC-22-0347 (PMC10035524; doi:10.1158/2767-9764.CRC-22-0347)
Supplement: Figure S6 — Dok-3 deficiency causes malignant progression of colorectal tumors of Apc mice. [file crc-22-0347-s08.pdf]

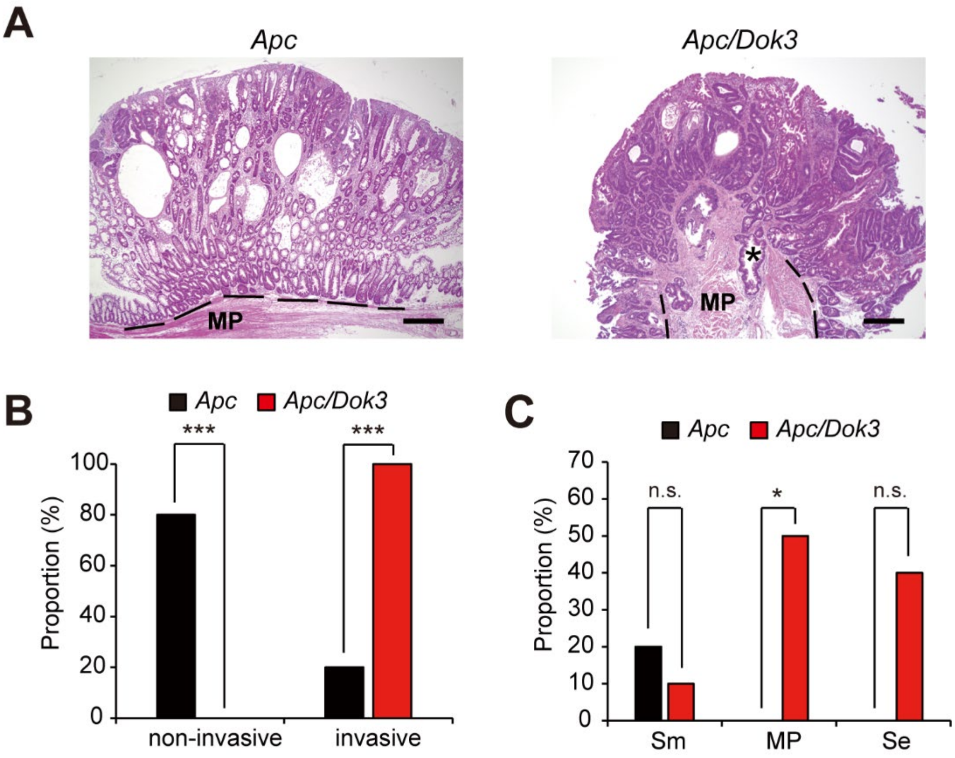

Supplementary Figure S6. Dok-3 deficiency causes malignant progression of colorectal tumors of *Apc* mice. (A) H&E-stained histological images of the colorectal tumors at 6 months of age. The dotted line shows the muscularis mucosae. The asterisk indicates a tumor penetrating beyond the muscularis propria (MP) and reaching the serosal surface. Scale bars, 200μm. (B) H&E-stained colorectal tumors ≥ 2 mm in diameter at 6-7 months of age were classified as non-invasive or invasive (as defined in Fig. 1D). The number of tumors classified as non-invasive or invasive is shown as proportions of the total tumor number analyzed (10 tumors from 3 *Apc* mice; 10 tumors from 3 *Apc/Dok3* mice). (C) Invasive tumors analyzed in B were further classified into 3 groups (Sm, MP, or Se as defined in Fig. 1E) and the number of tumors in each group is shown as a proportion of the total number of tumors (non-invasive and invasive) analyzed in B. **P* < 0.05; ****P* < 0.001 compared with *Apc* mice by Fisher's exact test. n.s., not significant.
