# Supplementary material for: Loss of Dok-3 in Non-tumor Cells Induces Malignant Transformation of Benign Epithelial Tumor Cells of the Intestine
Source: Cancer Res Commun. 2022 Dec 8;2(12):1590–600. doi: 10.1158/2767-9764.CRC-22-0347 (PMC10035524; doi:10.1158/2767-9764.CRC-22-0347)
Supplement: Figure S7 — mRNA expression of Dok-1 and Dok-2, but not Dok-3, is detectable in tumor epithelial cells. [file crc-22-0347-s09.pdf]

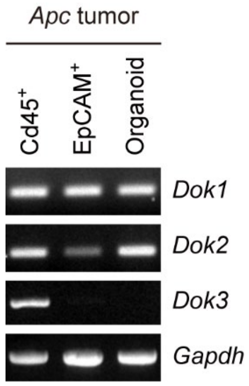


**Supplementary Figure S7. mRNA expression of *Dok-1* and *Dok-2,* but not *Dok-3*, is detectable in tumor epithelial cells.** Expression analysis of *Dok1/2/3* genes in leukocytes, epithelial cells and organoids. Leukocytes ($CD45^+$) and epithelial cells ($EpCAM^+$) from tumors in the small intestines of *Apc* mice were fractionated by flow cytometry. Organoids were generated from tumors in the small intestines of *Apc* mice. Total RNA from these cells and organoids were subjected to semiquantitative RT-PCR for each mRNA.
